# Efficacy of third-line anti-EGFR-based treatment versus regorafenib or trifluridine/tipiracil according to primary tumor site in RAS/BRAF wild-type metastatic colorectal cancer patients

**DOI:** 10.3389/fonc.2023.1125013

**Published:** 2023-02-21

**Authors:** Lisa Salvatore, Maria Bensi, Raffaella Vivolo, Ina Valeria Zurlo, Emanuela Dell’Aquila, Roberta Grande, Annunziato Anghelone, Alessandra Emiliani, Fabrizio Citarella, Maria Alessandra Calegari, Marta Ribelli, Michele Basso, Carmelo Pozzo, Giampaolo Tortora

**Affiliations:** ^1^ Oncologia Medica, Comprehensive Cancer Center, Fondazione Policlinico Universitario Agostino Gemelli, IRCCS, Rome, Italy; ^2^ Oncologia Medica, Università Cattolica del Sacro Cuore, Rome, Italy; ^3^ Oncologia Medica, Ospedale Fatebenefratelli Isola Tiberina - Gemelli Isola, Rome, Italy; ^4^ Department of Medical Oncology, Campus Bio-Medico University of Rome, Rome, Italy; ^5^ Medical Oncology 1, IRCCS Regina Elena National Cancer Institute, Rome, Italy; ^6^ UOSD Coordinamento Screening Oncologici, ASL Frosinone, Frosinone, Italy; ^7^ DH Oncologico, Ospedale F. Spaziani - ASL, Frosinone, Italy

**Keywords:** colorectal cancer, primary tumor site, third-line therapy, RAS/BRAF wild-type, anti-egfr ab, Regorafenib, trifluridine/tipiracil

## Abstract

**Background:**

Right- (R) and left-sided (L) metastatic colorectal cancer (mCRC) exhibit different clinical and molecular features. Several retrospective analyses showed that survival benefit of anti-EGFR-based therapy is limited to RAS/BRAF wt L-sided mCRC patients. Few data are available about third-line anti-EGFR efficacy according to primary tumor site.

**Methods:**

RAS/BRAF wt patients mCRC treated with third-line anti-EGFR-based therapy versus regorafenib or trifluridine/tipiracil (R/T) were retrospectively collected. The objective of the analysis was to compare treatment efficacy according to tumor site. The primary endpoint was progression-free survival (PFS); secondary endpoints were overall survival (OS), response rate (RR) and toxicity.

**Results:**

A total of 76 RAS/BRAF wt mCRC patients, treated with third-line anti-EGFR-based therapy or R/T, were enrolled. Of those, 19 (25%) patients had a R-sided tumor (9 patients received anti-EGFR treatment and 10 patients R/T) and 57 (75%) patients had a L-sided tumor (30 patients received anti-EGFR treatment and 27 patients R/T). A significant PFS [7.2 vs 3.6 months, HR 0.43 (95% CI 0.2-0.76), p= 0.004] and OS benefit [14.9 vs 10.9 months, HR 0.52 (95% CI 0.28-0.98), p= 0.045] in favor of anti-EGFR therapy vs R/T was observed in the L-sided tumor group. No difference in PFS and OS was observed in the R-sided tumor group. A significant interaction according to primary tumor site and third-line regimen was observed for PFS (p= 0.05). RR was significantly higher in L-sided patients treated with anti-EGFR vs R/T (43% vs. 0%; p <0.0001), no difference was observed in R-sided patients. At the multivariate analysis, third-line regimen was independently associated with PFS in L-sided patients.

**Conclusions:**

Our results demonstrated a different benefit from third-line anti-EGFR-based therapy according to primary tumor site, confirming the role of L-sided tumor in predicting benefit from third-line anti-EGFR vs R/T. At the same time, no difference was observed in R-sided tumor.

## 1 Introduction

The primary tumor site of metastatic colorectal cancer (mCRC) is associated with specific clinical-pathological and molecular features (1). From an anatomical point of view, differential characteristics between left- (L) and right- (R) sided tumors are based on embryological origin, physiological function, food transit, and gut microbiome (2). From a genetic and molecular point of view, R-sided colon cancer is associated with RAS and BRAF mutations and DNA mismatch-repair enzyme deficiency, while L-sided colon cancer is associated with EGFR, HER2-neu, APC, and TP53 mutations (3). Several studies demonstrated that the primary tumor site has both a prognostic and predictive role. Regarding the prognostic role, a metanalysis of 66 studies, including 1437846 mCRC patients, showed that L-sided tumor site was associated with longer OS in comparison to R-sided tumor site [HR 0.82 (95% CI 0.79-0.84), p< 0.001] (4). Regarding the predictive role, a metanalysis of 13 randomized controlled trials, investigated the correlation between efficacy of first-line therapy (bevacizumab vs anti-EGFR-based treatment) in mCRC patients and primary tumor location. In patients with RAS/BRAF wild-type (wt) L-sided mCRC, an anti-EGFR based first-line therapy showed an improved PFS and OS in comparison to bevacizumab-based treatment [PFS: HR 0.86 (95% CI 0.73-1.02); OS: HR 0.71 (95%CI 0.58-0.85)]. By contrast, in R-sided mCRC patients, the benefit from bevacizumab plus chemotherapy was higher as compared to anti-EGFR-based treatment [PFS: HR 0.65 (95%CI 0.50-0.86); OS: HR 0.77 (95%CI 0.57- 1.03)] (5). Accordingly, international and national guidelines (6, 7) recommend anti-EGFR plus chemotherapy for the first-line treatment of all wt L-sided mCRC patients as preferred option.

However, besides first-line treatment, few clinical data is available on the prognostic and/or predictive role of the primary tumor site for subsequent lines of therapy. With respect to anti-EGFR therapy efficacy for pretreated mCRC patients, Brule í et al.,reanalyzed the results of NCIC CO.17 trial (cetuximab vs best supportive care) according to primary tumor site. In this study, primary tumor location was not prognostic, but strongly predictive: KRAS wt L-sided mCRC patients had significantly longer PFS when treated with cetuximab compared to best supportive care [5.4 vs 1.8 months, HR 0.28 (95% CI 0.18-0.45), p < 0.0001], while no difference was observed in R-sided mCRC patients [1.9 vs 1.9 months, HR 0.73 (95%CI 0.42-1.27), p = 0.26] (interaction p=0.002) (8). Boeckx et al., in a retrospective analysis of study 20050181 (FOLFIRI-Panitumumab vs FOLFIRI) and study 20020408 (panitumumab vs best supportive care), investigated the efficacy of anti-EGFR-based therapy, after first-line, according to primary tumor location. RAS wt L-sided tumor had better outcomes with panitumumab than with the comparator treatment [study 20050181 PFS: 8.0 vs 5.8 months, HR 0.88 (95% CI 0.69-1.12), p=0.31, study 20020408 PFS: 5.5 vs 1.6 months, HR 0.50 (95% CI 0.22-1.15), p< 0.0001] (9).

To date, regorafenib (R) and trifluridine/tipiracil (T) represent two standard treatment options for chemorefractory mCRC patients. In the CORRECT (10) and RECOURSE (11) trials, R and T showed a significant OS improvement in comparison to best supportive care [HR 0,77 (IC 95% 0,64-0,94) p 0,0052] [HR 0.66 (IC 95% 0.56-0.78), p<0.001], respectively. Despite the statistically significant OS improvements, the absolute benefit appeared limited, and it was independent from both RAS status and primary tumor site.

Based on this limited evidence, we retrospectively compared the efficacy of third-line therapy with anti-EGFR-based treatment versus R/T in RAS/BRAF wt mCRC patients, according to the primary tumor site.

## 2 Materials and methods

### 2.1 Study population

Patients with RAS and BRAF wt mCRC, treated with R or T versus anti-EGFR-based treatment in third-line, were retrospectively included in the study. Patients were enrolled by four Italian Medical Oncology Units (Comprehensive Cancer Center, Fondazione Policlinico Universitario Agostino Gemelli-IRCCS, Università Cattolica del Sacro Cuore, Rome; Ospedale Fatebenefratelli Isola Tiberina - Gemelli Isola, Rome; Department of Medical Oncology, Campus Bio-Medico University, Rome; Ospedale F. Spaziani - ASL Frosinone)

Patients had to have received two prior regimens of standard chemotherapy (oxaliplatin, irinotecan, fluoropyrimidine) for metastatic disease. Previous treatments could include bevacizumab. Patients who received cetuximab and/or panitumumab in first- or second-line were excluded from the anti-EGFR group; on the contrary, they could be enrolled in the R/T group. Prior therapy with R or T was not allowed.

The R-sided tumor was defined as cancer from the cecum to the transverse colon, L-sided tumor was defined as cancer from the splenic flexure to the rectum. For each patient we collected the following available variables: baseline ECOG performance status (PS), gender, age, synchronous vs metachronous disease, previous anticancer treatments, and number of metastatic sites (single vs multiple).

### 2.2 Study outcomes

This is a retrospective, multicenter, observational study aiming to investigate the predictive role of primary tumor site in RAS/BRAF wt mCRC patients receiving anti-EGFR or R/T as third-line treatment. The primary endpoint was progression-free survival (PFS); the secondary endpoints were overall survival (OS), response rate (RR), and toxicity. PFS was defined as the time from the start of third-line treatment to disease progression or death from any cause, whichever occurred first. OS was defined as the time from treatment start to the date of death for any reasons. RR was the percentage of patients achieving an objective response (complete response or partial response) according to RECIST criteria (version 1.1). Disease evaluation was performed with a computed tomography (CT) scan of chest and abdomen every 8-12 weeks, according to clinical practice. Toxicity rate was defined as the percentage of patients experiencing a specific adverse event (AE) during the treatment, according to NCTCAE version 5.0.

### 2.3 Statistical analysis

Chi-square test was performed to compare patient characteristics and RR between R- and L- tumor groups, and incidence of AEs according to treatment group. PFS and OS analyses were carried out using the Kaplan-Meier method. Cox proportional regression was used for univariate and multivariate analyses of PFS and OS. Statistical significance was established at p = 0.05. Hazard ratios (HR) with 95% confidence interval (CI) were estimated using a logistic regression model. All analyses were conducted using MedCalc statistical software version 18.11.3 (MedCalc Software, Ostend, Belgium; http://www.medcalc.org;2019).

## 3 Results

### 3.1 Patients characteristics

A total of 76 RAS/BRAF wt mCRC patients, receiving, as third-line treatment, R or T or anti-EGFR based-therapy, were enrolled in the study. Fifty-seven (75%) patients had a L-sided tumor, 19 (25%) patients had a R-sided tumor. Thirty-nine (51%) patients received anti-EGFR-based therapy (16 patients panitumumab and 23 irinotecan plus cetuximab), 37 (49%) patients received R or T. Among patients with L-sided tumor, 30 (53%) were treated with anti-EGFR-based therapy and 27 (47%) with R/T. Among patients with R-sided tumor, 9 (47%) were treated with anti-EGFR-based therapy and 10 (53%) with R/T.

Baseline clinical characteristics were well-balanced between the two groups. The median age was 64 years (range 38-81) in the L-sided tumor group, and 63 years (range 38-78) in the R-sided tumor group. Males were 51% and 53% in the L- and R-sided tumor group, respectively; ECOG PS was 0 in 28% and 26%; metastases were synchronous in 72% and 79%; sites of metastases were multiple in 72 and 74% of L- and R-sided tumor group, respectively. Clinical baseline patients characteristics and treatment information are summarized in **Table 1**.

**Table 1:** Patients characteristics.

Characteristics, N (%)	Right-sided (N = 19)	Left-sided (N = 57)	p-value
Age (years), median (range)	63 (38-78)	64 (38-81)	
≤65 years	13 (68.4)	30 (52.6)	0.11
>65 years	6 (31.6)	27 (47.4)	
Sex			
Male	10 (52.6)	29 (50.9)	0.89
Female	9 (47.4)	28 (49.1)	
ECOG PS at the beginning of 3^rd^ line			
0	5 (26.3)	16 (28)	0.88
1-2	12 (63.2)	35 (61.4)	
NA	2 (10.5)	6 (10.6)	
Time between diagnosis of PT and metastases			
Synchronous (≤ 6 months)	15 (78.9)	41 (72)	0.55
Metachronous (> 6 months)	4 (21.1)	16 (28)	
3^rd^ line therapy			
Anti-EGFR	9 (47.4)	30 (52.6)	0.69
R/T	10 (52.6)	27 (47.4)	
N metastatic sites at the beginning of 3^rd^ line			
1	5 (26.3)	16 (28.1)	0.88
≥2	14 (73.7)	41 (71.9)	
Prior systemic anticancer agents			
fluoropyrumidine	19 (100)	57 (100)	1
oxaliplatin	15 (78.9)	53 (92.9)	0.08
irinotecan	18 (94.7)	56 (98.2)	0.41
bevacizumab	14 (73.7)	40 (70.2)	0.77
Anti-EGFR	10 (52.6)	26 (45.6)	0.59

N, number; ECOG PS, Eastern Cooperative Group Performance Status; PT, primary tumor; NA, not applicable; R/T, Regorafenib or Trifluridine/Tipiracil.

### 3.2 Efficacy and activity of third-line treatment according to primary tumor site

**Figure 1:**
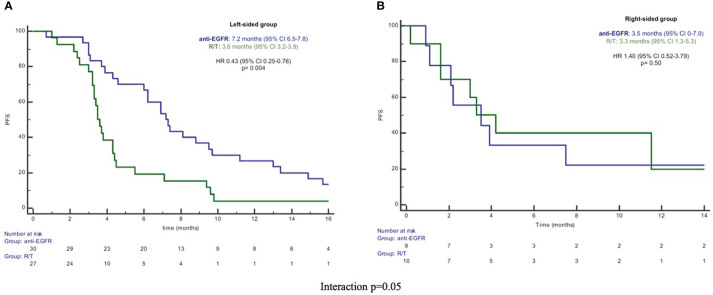
**(A)** Kaplan-Meier PFS curves in left-sided group. **(B)** Kaplan-Meier PFS curves in right-sided group. PFS, progression-free survival; R/T, Regorafenib or Trifluridine/Tipiracil; HR, hazard ratio; CI, confidence interval.

**Figure 2:**
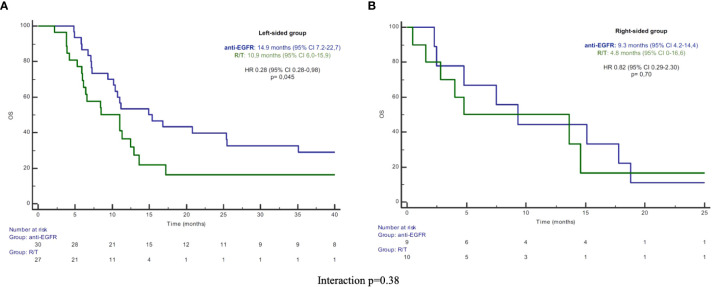
**(A)** Kaplan-Meier OS curves in left-sided group. **(B)** Kaplan-Meier OS curves in right-sided group. PFS, progression-free survival; R/T, Regorafenib or Trifluridine/Tipiracil; HR, hazard ratio; CI, confidence interval.

In the L-sided tumor group, median PFS and OS were significantly longer in patients treated with anti-EGFR in comparison to patients treated with R/T [median PFS: 7.2 (95% CI 6.5-7.8) vs 3.6 months (95% CI 3.2-3.9), HR 0.43 (95% CI 0.2-0.76), p=0.004; median OS: 14.9 (95% CI 7.2-22.7) vs 10.9 months (95% CI 6.0-15.9), HR 0.52 (95% CI 0.28-0.98), p=0.045]. By contrast, in the R-sided tumor group, no significant difference in both PFS and OS according to treatment was observed [median PFS: 3.5 (95% CI 0-7.0) vs 3.3 months (95% CI 1.3-5.3), HR 1.40 (95% CI 0.52-3.79), p=0.50; median OS: 9.3 (95% CI 4.2-14.4) vs 4.8 months (95% CI 0-16.0), HR 0.82 (95%CI 0.29-2.30), p=0.70] (**Figures 1**, **2**). A significant interaction according to primary tumor site and third-line regimen was observed for PFS (p=0.05), but not for OS (p=0.38) (**Figure 1**, **2**).

**Figure 3:**
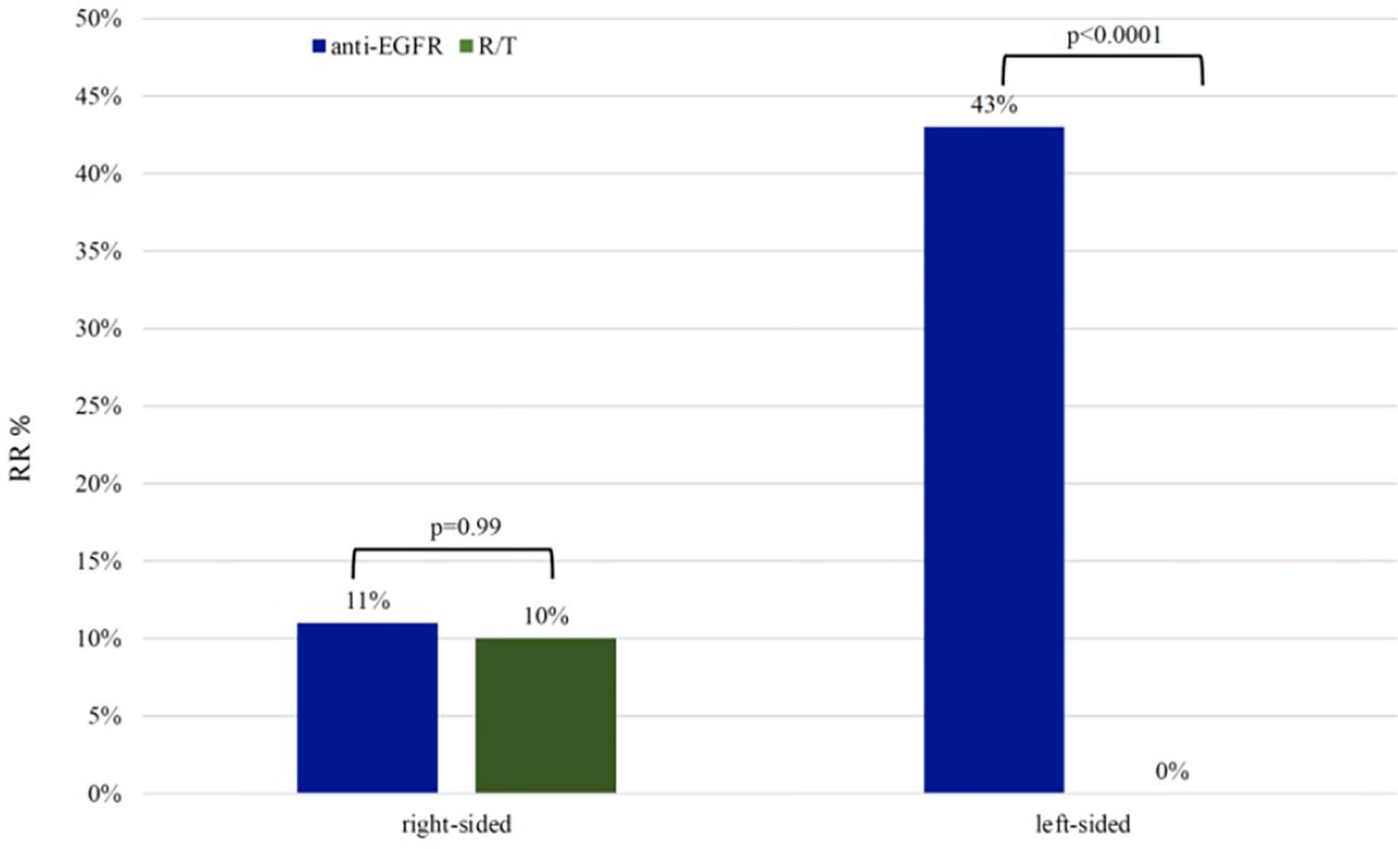
Response rate in left-sided group and right-sided group RR, response rate; R/T, Regorafenib or Trifluridine/tipiracil.

In the L-sided tumor group, RR was 43% in patients treated with anti-EGFR and 0% in patients treated with R/T (p <0.0001). No difference in RR was observed in patients with R-sided colon cancer according to treatment (RR 11% in patients treated with anti-EGFR vs RR 10% in patients treated with R/T, p=0.99) (**Figure 3**).

At the multivariate analysis, in the L-sided tumor group, third line regimen (anti-EGFR vs R/T) was independently associated with PFS [HR 0.45 (95% CI 0.25–0.80), p=0.006], but not with OS. By contrast, in the R-sided tumor group, at the multivariate analysis no association between third-line regimen and survival outcomes was observed. Univariate and multivariate analyses for PFS and OS are showed in **Table 2**.

**Table 2:** Univariate and multivariate analyses for PFS and OS.

Variables	PFS				OS			
	Univariate Analysis HR (95% CI); p-value		Multivariate Analysis HR (95% CI); p-value		Univariate Analysis HR (95% CI); p-value		Multivariate Analysis HR (95% CI); p-value	
	Right-sided	Left-sided	Right-sided	Left-sided	Right-sided	Left-sided	Right-sided	Left-sided
Median age ≤65 vs >65 years	0.92 (0.32–2.60); *p* = 0.87	0.98 (0.58–1.68); *p* = 0.95	–	–	1.28 (0.44-3.67); p=0.65	1.26 (0.70–2.26); *p* = 0.44	–	–
N metastatic sites at the beginning of 3^rd^ line 1 vs ≥2	0.07 (0.01–0.55); *p* = 0.01	0.46 (0.25–0.86); *p* = 0.01	0.07 (0.01-0.55); *p* = 0.01	0.48 (0.26–0.90); *p* = 0.02	0.10 (0.01-0.80); p=0.03	0.25 (0.11–0.56); *p* = 0.001	–	0.25 (0.11–0.56); *p* = 0.001
Time between diagnosis of PT and metastases Synchronous vs metachronous	8.36 (1.07–65.36); *p* = 0.043	1.02 (0.56–1.87); *p* = 0.95	–	–	10.93 (1.37-87.31); *p* = 0.02	1.20 (0.63–2.29); *p* = 0.58	10.93 (1.37-87.31); p = 0.02	–
3^rd^ line therapy Anti-EGFR vs R/T	1.40 (0.52–3.79); *p* = 0.50	0.43 (0.25– 0.76); *p* = 0.004	–	0.45 (0.25–0.80); *p* = 0.006	0.82 (0.29–2.30); *p* = 0.70	0.52 (0.28–0.98); *p* = 0.045	–	–

PFS, progression-free survival; OS, overall survival; N, number; HR, hazard ratio; CI, confidence interval; PT primary tumor R/T, Regorafenib or Trifluridine/Tipiracil.

### 3.3 Toxicity

The incidence of any grade and grade 3/4 AEs was significantly higher in patients treated with R/T in comparison to patients treated with anti-EGFR (any grade: 88% vs 64%, p=0.018; grade 3/4: 47% vs 20%, p=0.017).

The most frequent AE in patients treated with anti-EGFR was folliculitis (any grade 49%, grade 3/4 13%), while the most frequent AEs in patients treated with R/T were hand-foot syndrome (any grade 35%, grade 3/4 6%), hypertension (any grade 24%, grade 3/4 6%), neutropenia (any grade 21%, grade 3/4 15%) and anemia (any grade 12%, grade 3/4 6%]. The incidence of AES was reported in **Table 3**.

**Table 3:** Adverse Events.

Adverse events	Anti-EGFR (N=39)	R/T (N=34)	p- value	Anti-EGFR (N=39)	R/T (N=34)	p- value
	Any grade N (%)	Any Grade N (%)		Grade 3-4 N (%)	Grade 3-4 N (%)	
Any adverse events	25 (64)	30 (88)	0.018	8 (20%)	16 (47)	0.017
Fatigue	10 (26)	16 (47)	0.06	1 (3%)	3 (9)	0.24
Nausea	1 (3)	3 (9)	0.24	0 (0)	0 (0)	1
Diarrhea	5 (13)	5 (15)	0.89	0 (0)	1 (3)	0.86
Stomatitis	5 (13)	3 (9)	0.59	0 (0)	0 (0)	1
Dermatitis acneiform	19 (49)	0 (0)	<0.00001	5 (13)	0 (0)	0.02
Hand-foot syndrome	2 (5)	12 (35)	0.001	0 (0)	6 (18)	0.02
Hypertension	0 (0)	8 (24)	0.005	0 (0)	2 (6)	0.03
Neutropenia	4 (10)	7 (21)	0.22	2 (5)	5 (15)	0.16
Anemia	0 (0)	4 (12)	0.12	0 (0)	3 (9)	0.24
Trombocytopenia	2 (5)	0 (0)	0.46	0 (0)	0 (0)	1
Transaminases increase	0 (0)	2 (6)	0.461	0 (0)	0 (0)	1

N, number; R/T, Regorafenib or Trifluridine/tipiracil.

## 4 Discussion

To the best of our knowledge, this study was the first investigating the efficacy of a third-line therapy with anti-EGFR-based treatment versus R/T in RAS/BRAF wt mCRC patients, according to the primary tumor site. Our results confirm the benefit of third-line anti-EGFR treatment in L-sided tumors, supporting the predictive role of primary tumor location also in pretreated mCRC patients.

The benefit of first-line chemotherapy plus cetuximab or panitumumab in L-sided mCRC has been clearly demonstrated (5), while clinical evidence on the role of primary tumor site in predicting benefit from EGFR inhibitors in pretreated mCRC patients is still limited. Chen et al., in a cohort study of 969 KRAS wt mCRC patients treated with third-line cetuximab, demonstrated a significant longer time to treatment discontinuation (p=0.0005) and OS (p <0.0001) in L-sided vs R-sided tumor patients, confirming the prognostic role of primary tumor site (12). Moretto et al., analyzing 75 RAS/BRAF wt mCRC patients treated with cetuximab +/- irinotecan or panitumumab as first-line or subsequent lines, demonstrated a lack of activity of anti-EGFR in R-sided vs L-sided tumors. Specifically, RR was 0% and 41% in R-sided and L-sided tumor patients (p=0.0032), respectively (13). The main limitations of these studies are the retrospective nature and the lack of a control arm.

Concerning treatment with R/T, the impact of the primary tumor site was not well defined. Subgroup analyses of both CORRECT and RECOURSE trials demonstrated a survival benefit regardless of primary tumor site (10, 11). In a multicenter retrospective study of 505 mCRC patients treated with R or T, R-sided patients had a shorter OS in comparison to L-sided patients (p=0.041), but at the multivariate analysis for OS, primary tumor location was not an independent prognostic factor (p=0.64) (14).

The strength of our study was stringent inclusion criteria for patients: we selected only RAS/BRAF wt mCRC patients, also in the R/T group, in order to evaluate a homogeneous population; previous treatment with cetuximab or panitumumab was not allowed in the anti-EGFR group, thus avoiding potentially resistant patients. Furthermore, our retrospective study, compared third-line anti-EGFR therapy with R/T, a standard treatment option in pretreated mCRC patients.

Our study population was characterized by an imbalance in the primary tumor site (75% L-sided side vs 25% R-sided), that could be explained by the different molecular profiling between L- and R-sided tumors. Our analysis showed a significant longer PFS and OS for patients treated with anti-EGFR vs R/T in the L-sided tumor group [median PFS 7.2 vs 3.6 months, HR 0.43 (95% CI 0.2-0.76), p=0.004; median OS 14.9 vs 10.9 months, HR 0.52 (95% CI 0.28-0.98), p=0.045]. By contrast, no significant difference in survival outcomes was observed between anti-EGFR vs R/T in the R-sided tumor group [median PFS 3.5 vs 3.3 months, HR 1.40 (95% CI 0.52-3.79), p=0.50; median OS 9.3 vs 4.8 months, HR 0.82 (95%CI 0.29-2.30), p=0.70]. A significant interaction according to primary tumor site and third-line treatment was observed for PFS (p 0.05). In the multivariate analysis, the third-line regimen was independently associated with PFS [HR 0.45 (95% CI 0.25–0.80), p=0.006] in the L-sided tumor group. Also, regarding the activity, we observed a different RR according to third-line regimen and primary tumor site: in particular, in the L-sided tumor group, RR was 43% in patients treated with anti-EGFR and 0% in patients treated with R/T (p <0.0001), while no difference was observed in the R-sided tumor group. Our results confirmed the predictive role of the primary tumor site for third-line anti-EGFR-based treatment in RAS/BRAF wt patients.

The different distribution of consensus molecular subtypes (CMS) between L- and R-sided tumors may explain the different sensitivity to anti-EGFR according to primary tumor site. L-sided tumors are more representative of CMS2, enriched for epithelial signature, and CMS4, associated to epithelial-mesenchymal transition (3, 15, 16). CMS2 is an over-activated epithelial growth factor pathway with higher expression of EGFR and the EGFR-ligands amphiregulin and epiregulin, that are correlated to an increased response to EGFR inhibitor therapy in RAS/BRAF wt CRC (17). Stintzing et al., analyzing gene signature of 514 samples of patients enrolled in the FIRE-3 study, demonstrated that patients with CMS4 tumors had a longer OS when treated with cetuximab vs bevacizumab (18). In another molecular analysis of RAS/BRAF wt patients from the COIN and PICCOLO study, patients with CMS4 tumors showed a a longer OS and PFS when treated with anti-EGFR-based treatment vs chemotherapy alone (19).

The different benefit from anti-EGFR according to primary tumor site in RAS/BRAF wt mCRC patients may be also explained by a heterogeneity of primary resistance profile. Not only the well-known mutations in RAS and BRAF genes, but also the less common alterations such as HER2 and MET amplification, deregulation of the PI3K/PTEN/AKT axis, NTRK/ROS/ALK/RET rearrangements, may represent negative predictive factors for response to anti-EGFR (20). Morano et al., analyzing RAS/BRAF wt mCRC patients receiving panitumumab-based maintenance therapy in the Valentino trial, demonstrated that the combined assessment of sidedness and molecular alterations of primary resistance to anti-EGFR according to PRESSING panel (21) identified a subpopulation with inferior benefit from anti-EGFR-based therapy (22).

Concerning the safety profile, our study, showed a significant higher incidence of AEs in the group of patients treated with R/T in comparison to anti-EGFR-based therapy (p 0.018), and a drug-specific toxicities (hand-foot syndrome and hypertension for R, neutropenia and anemia for T, folliculitis for anti-EGFR), as previously reported.

Our study presented several limitations, such as the retrospective design, the lack of randomization, the lack of a negative hyperselection, such as with the PRESSING panel, and the small sample size, especially for the R-sided group. We did not explore the optimal therapeutic sequence, as investigated by the REVERCE trial, which reported a longer OS for patients receiving regorafenib followed by cetuximab vs the reverse sequence [17.4 vs 11.6 months, HR 0.61 (95% CI 0.39–0.96), p 0.0293] (23). Furthermore, our study excluded patients receiving anti-EGFR rechallenge according to CRICKET (24) and CHRONOS trials (25). The ongoing randomized PARERE study (26), investigating rechallenge with panitumumab followed by regorafenib versus the reverse sequence in chemorefractory RAS/BRAF wt patients selected by liquid biopsy, could further clarify the role of anti-EGFR according to primary tumor site.

## 5 Conclusion

In conclusion, our results demonstrated a different benefit from third-line anti-EGFR therapy according to primary tumor site, confirming the role of L-sided tumor in predicting benefit from third-line anti-EGFR vs R/T. At the same time, no difference was observed in R-sided tumors. Despite several limitations, our study confirmed previous evidence and, waiting for results from the PARERE trial, we can conclude that the preferred third-line option for RAS-BRAF wt L-sided mCRC patients, not yet treated with panitumumab or cetuximab, is still anti-EGFR. By contrast, in R-sided mCRC patients, the choice between anti-EGFR and R/T should be based on previous treatment toxicity and patient clinical conditions.

## Data availability statement

The raw data supporting the conclusions of this article will be made available by the authors, without undue reservation.

## Author contributions

LS and MBe contributed to conception, design of the study, organized the database, performed the statistical analysis and wrote the manuscript. RV, IZ, ED, RG, AA, AE, FC, MC, MR, MBa, CP involved in the data collection. GT contributed to manuscript revision, read, and approved the submitted version. All authors contributed to the article and approved the submitted version.
